# Science and Surgery

**Published:** 1889-03-30

**Authors:** 


### WITHIN THE WARDS.

### SCIENCE AND SURGERY.

It is eminently desirable that the general public should possess itself of sufficient knowledge of the medical and allied sciences to enable it to understand and value justly the progress and state of medicine at any given period. How otherwise is it to be able to distinguish between capable and incapable practitioners, or between the claims of orthodox medicine and those of heterodoxy and quackery? The most enlightened practitioners of medicine themselves see that the worst enemies of their noble profession are those who would make it a mystery and an occult art, the secrets of which are to be rigidly kept within the limited circle of the initiated. The following case is one of deep interest to medical and other men of science, and may be readily understood and valued by any reader of ordinary intelligence. Some months ago W. M., a well-grown boy of nine, was taken to a London hospital, having been stunned by a fall from a cart. He had apparently struck his head either against the ground or some other hard object. His symptoms were very marked, and some of them decidedly peculiar. He vomited profusely, and seemed to be unable to bear the light, turning his eyes away from it as much as possible. He was, besides, very drowsy. When spoken to he made one answer, and one only. He constantly said, "Go away." The following day his state had so far changed that he could occasionally answer a question rationally, but he was extremely restless, muttered frequently, and kept jumping out of bed. Two days later, under medicinal treatment, he was so far improved that he had lost his restlessness and could apparently understand what was said to him, although he could not be got to answer questions. Speech, in fact, was paralysed. The slight improvement continued for two days longer, and then followed a series of symptoms which left no doubt that a dangerous injury to the head had taken place. At seven o'clock in the morning convulsions set in, and the first fit lasted half an-hour. Violent and tumultuous twitchings of the right side of the face and neck were the principal characteristics of this fit. At half-past eight a second fit seized the boy, and on this occasion the eyes and mouth were drawn to the right side, the tongue worked backwards and forwards periodically, and the right fist was firmly clenched. He had nine such fits during the day, and at the end of that time the muscles of the right side of the face and the right arm seemed to be worn out. During the following day the fits were slighter and not very numerous, but towards evening a most violent attack came on and lasted nearly two hours; not only were the face, neck, mouth, and eyes all in a state of constant spasmodic movement, but hiccough set in, the wrist worked violently, and the fingers of the right hand were fearfully contracted about ninety times a minute. These systems continuing without any sign of remission, it was decided that an operation should be performed. But what was to be done? and where? Judging from the symptoms, the surgeon in charge was able to say with a degree of certainty that some kind of pressure was being exerted on a particular portion of the brain, which he indicated. Ferrier, as the result of his experiments, has mapped out with considerable exactitude those parts of the brain which supply with energy different sets of muscles. If a set of muscles, say of the arm, or face, or leg, begins to work independently of the will, it is concluded that there is a cause for the uncontrolled movements, and that the cause is to be looked for in that part of the brain or spinal cord which supplies the defaulting muscles with their nerve force. Accordingly, the surgeon decided that an effusion of blood was pressing upon a defined portion of the brain. At the spot indicated the soft parts were cut through, a circular piece of bone, an inch in diameter, was sawn out, and the brain exposed. The hard outer covering which first appeared was removed, and there was the blood exactly as had been predicted. The fluid portion flowed out of the opening, and a firmer fixed clot then became visible. In order to remove all the clot without breaking it and leaving a piece behind, wh ch wou d have only been half a cure, another portion of bone was removed by the trephine, and the clot taken away. It was estimated to consist of half a fluid ounce of blood — four small teaspoonfuls. By means of a soft instrument passed into the space where the clot had been, the cavity was washed clean out with warm carbolised water. The case should now have been complete; but, unfortunately after the wound had been dressed, and the boy had remained quiet for some time, the fits returned, and all power of speech remained lost. This continuing for two days, it was resolved to reopen the wound to see if fresh blood had not flowed since the operation. The result proved that the suspicions of the surgeons were correct. All the newly extravasated blood was removed, the parts were washed out as before, and the wound again closed. The result was in all respects happy. The fits diminished both in frequency and violence, and finally ceased. On the second day the boy could shake hands with the right hand, which had formerly been helpless; three days later he could articulate a few words when questioned. Finally the power of speech returned to its normal condition, and every other unfavourable symptom disappeared. The boy was quite well If this case had been left to nature, abscess of the brain would probably have developed, and the child would have died in agony. In any event a cure without operation was so unlikely as to be practically impossible. Such a case is one of the undoubted scientific triumphs of modern surgery.

